# Hard and soft tissue shape variation and changes in Class II division 1 malocclusion during orthodontic treatment: a geometric morphometric analysis

**DOI:** 10.1186/s12903-023-03684-7

**Published:** 2023-11-27

**Authors:** Chin Sin Chu, Murshida Marizan Nor, Alizae Marny Mohamed, Helmi Mohd Hadi Pritam

**Affiliations:** 1 Department of Family Oral Health, Faculty of Dentistry, Universiti Kebangsaan Malaysia, Jalan Raja Muda Abdul Aziz, Kuala Lumpur, 50300 Malaysia; 2 School of Health Sciences, USM Health Campus, Kelantan, Malaysia

**Keywords:** Class II division 1, Geometric Morphometric, Soft tissue, Treatment changes

## Abstract

**Background:**

This study aims to determine the hard and soft tissue shape variation and its changes in Class II division 1 malocclusion before and after orthodontic treatment using Geometric Morphometric Analysis.

**Methods:**

This retrospective study included 141 pre-treatment and near-end treatment lateral cephalometric radiographs of Class II division 1 malocclusion patients aged 16–40 years with a skeletal II pattern (ANB > 4^o^). 32 landmarks in Cartesian coordinates were created and identified using MorphoJ software to establish a shape analysis.

**Results:**

The vertical dimensions (hypodivergent to hyperdivergent facial profiles) showed the largest variation in the general shape of hard and soft tissue, followed by the anteroposterior dimensions (mild to severe skeletal II patterns). Variations of lip shape (long to short), lip protuberance (everted to inverted), and nasolabial angle (obtuse to acute) were present. Orthodontic treatment affected the shape of the hard and soft tissue significantly (*p* < 0.0001). T2 showed significant uprighting of upper incisors (17.5^o^) and lower incisors (3.7^o^), improved NLA (8^o^), an increase in upper lip thickness (1.5 mm), and a reduction in lower lip thickness (0.7 mm) (*p* < 0.05).

**Conclusion:**

Vertical and anteroposterior shape variations were found. Orthodontic treatment had an impact on both hard and soft tissue shapes. Hence, understanding both the hard and soft tissue shape variations and the orthodontic treatment changes is crucial for an accurate diagnosis and treatment plan to achieve a successful outcome and excellent patient satisfaction.

**Supplementary Information:**

The online version contains supplementary material available at 10.1186/s12903-023-03684-7.

## Background

The prevalence of Class II division 1 malocclusion is the highest after Class I malocclusion and is one of the most common malocclusion seeking orthodontic treatment in an orthodontic clinic [1]. Skeletally, patients could present with a prognathic maxilla, a retrognathic mandible, or a combination of both [2]. Although they are usually associated with an increased facial angle and increased ANB, variation in the craniofacial vertically and horizontally occurs [3]. The upper and lower incisors of this malocclusion could be proclined or uprighted. The soft tissue could show protruded and incompetent lips due to underlying skeletal discrepancies and proclined upper incisors [4]. Understanding the underlying anteroposterior and vertical skeletal variations, dentoalveolar discrepancies, and the soft-tissue drape is paramount for orthodontic treatment plan as it may influences the anchorage demand [5], extraction pattern [6], as well as the orthodontic mechanics [7]. Each patient’s craniofacial morphologies are unique; thus, the treatment plan should be customised to achieve ideal occlusal, functional relationships, and aesthetics. In addition, patients are now often more concerned with their facial aesthetics, especially their smile [8]. Studies have shown that orthodontic treatment have a significant impact on soft tissue changes, which may affect the overall facial appearance and function [8]. Extraction may cause a decrease in the upper and lower lips prominence, a reduction in interlabial gaps, and an increase in the nasolabial angle. However, in non-extraction, the upper and lower lips may protrude slightly, and the lip thickness might decrease [9]. Without anticipating and discussing these changes in advance, treatment outcomes might be overestimated or procedural complexities might be underestimated. Utilizing a decent illustration can help manage patients’ expectations, provide a clear picture of achievable end-treatment results, and highlight the importance of orthodontic treatment compliance and relapse risks [10].

Conventional lateral cephalometric radiographs can quantitatively describe and analyse facial forms using linear distances, angles, and ratios. However, it is unable to adequately describe the craniofacial morphology, distinguish changes in curvature, and describe size, shape, and growth [11–13]. Multiple superimpositions are also impossible, making graphical shape analysis challenging and limiting its illustrations [14]. Geometric Morphometric Analysis (GMA) is an innovative statistical method that analyses anatomical landmarks quantitatively using Cartesian coordinates. It is commonly used to comparatively study the relationships between different shape components or groups [15].

Recently, GMA has been increasingly utilised in the orthodontics field. Knigge et al. [11] studied the growth of the hard tissue shapes in different facial types and found that hyperdivergent growth in patients is associated with Class II malocclusion. Woon et al. [12] studied the hard tissue shapes and found considerable variations in skeletal shapes. Sazgar et al. [16] studied the soft tissue differences in different sagittal skeletal patterns and concluded that different soft tissue shapes vary in different skeletal patterns. GMA has been used to study the shapes of the mandibular symphysis [17], arch forms [18], palatal rugae [19], and cleft patients [20].

Nevertheless, some studies using lollipop and wireframe graphs for shape illustration may not accurately replicate shape differences. Based on current updates, no studies have ever been done on hard and soft tissue shape changes during orthodontic treatment. The hypothesis has been that no hard and soft tissue shape variations or significant shape changes exist after orthodontic treatments. Therefore, this study aims to investigate the shape variations and changes of both hard and soft tissue in a Class II division 1 malocclusion before and after orthodontic treatment. This study could provide information on the shape variations of Class II division 1 malocclusion and provide graphical illustrations of the orthodontic treatment effects on the maxilla, mandible, and soft tissues, including their relationships with each other.

## Methods

### Samples

This retrospective study looked at the lateral cephalometric radiographs (LCR) and hard and soft tissue shapes and sizes in Class II division 1 malocclusion pre- and near-end-orthodontic treatment. All patients have completed orthodontic treatment in the Department of Family Oral Health, Universiti Kebangsaan Malaysia. The inclusion criteria for subjects were as follows: Age 16–40 years old, Class II division 1 malocclusion, medically fit and healthy, presence of all incisors, excellent LCR quality with all hard and soft tissue landmarks identifiable, and a skeletal II pattern (ANB > 4^o^). This study excluded patients with poor LCR, craniofacial or dental anomalies, clefts, a history of trauma or surgery to the mandible, a history of periodontitis, a history of previous orthodontic treatment, and missing incisors.

No clear guidelines exist for calculating sample size in a geometric morphometric analysis, and no straightforward mathematical formula exists to determine it [17]. In general, a minimum sample size within a group was suggested by using the number of coordinates and landmarks. Therefore, a study on two groups with 32 landmarks and 2D coordinates (x, y) requires 124 samples (32 × 2 × 2 = 124). Nevertheless, a sample size calculation was still made for linear measurements. To achieve a power of 80% and a 5% significance level for detecting the mean difference of 2.33 between pairs with a standard deviation of 6.62, a minimum of 66 samples was required [21]. Finally, this study included 141 samples in anticipation of dropouts or sample elimination due to poor LCR quality, but at the trial’s conclusion, all 141 samples were retained.

### Lateral cephalometric radiograph analysis

Pre-(T1) and near-end-(T2) treatment LCR were retrieved from the Department of Radiology at Universiti Kebangsaan Malaysia. Using Planmeca Romexis software (Planmeca ProMax 3D Classic; Planmeca Romexis, Helsinki, Finland), 141 LCR were obtained from both pre- and near-end treatment. The exposure parameters were 60–90 kV, 1–14 mA, an exposure time between 9 and 37 s, and a distance of 1.5 m between the source and the midsagittal plane. The LCR were digitally traced using VistaDent OC (2D) (GAC International, Inc., Bohemia, NY, USA). To ensure accurate and consistent LCR tracing, all radiographs were rotated in such a way that Frankfort horizontal plane is used as the true horizontal plane, as a reference line to define the precise location of all other landmarks.

**Fig. 1:**
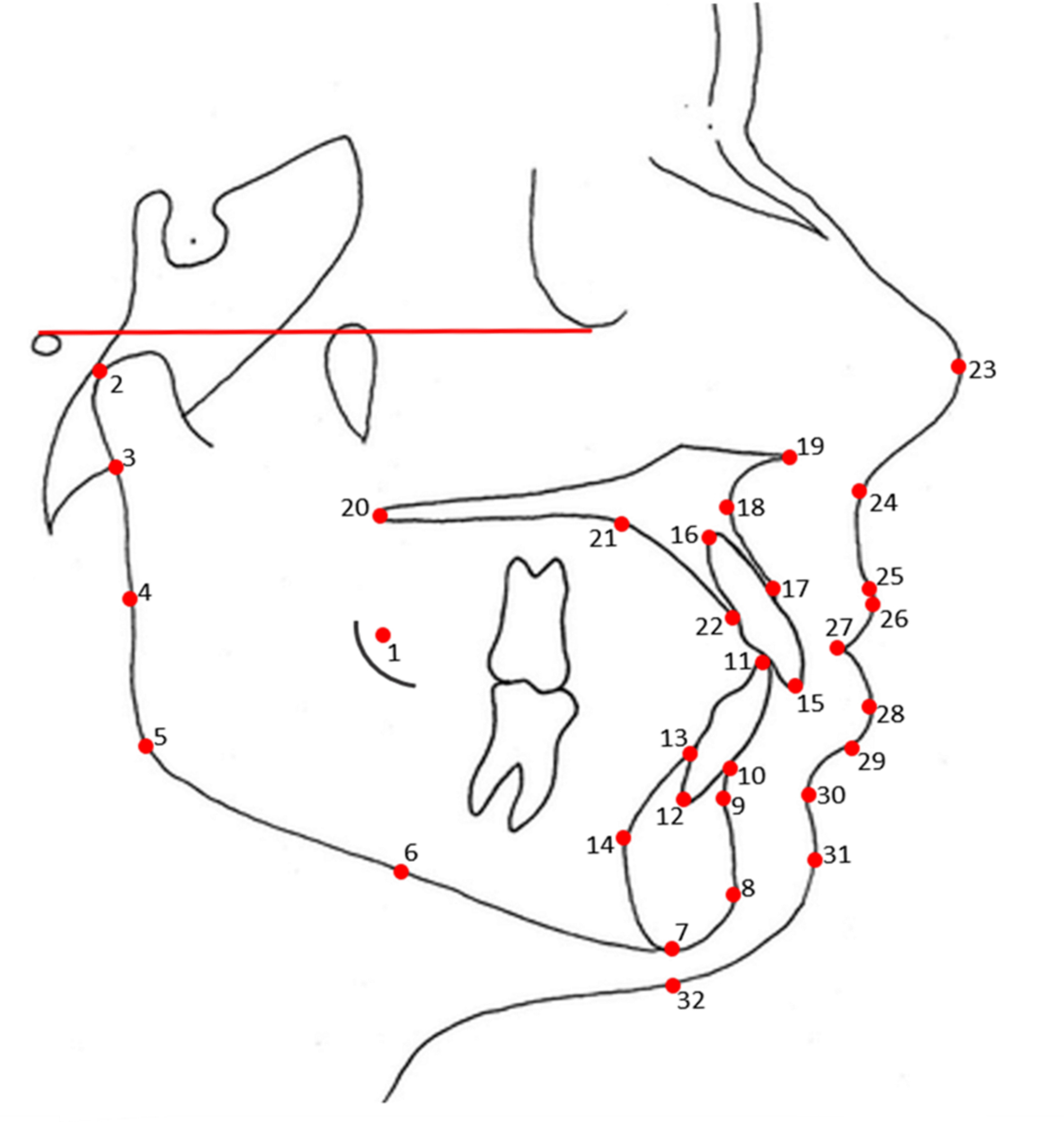
Landmarks for Geometric Morphometric Analysis

**Fig. 2:**
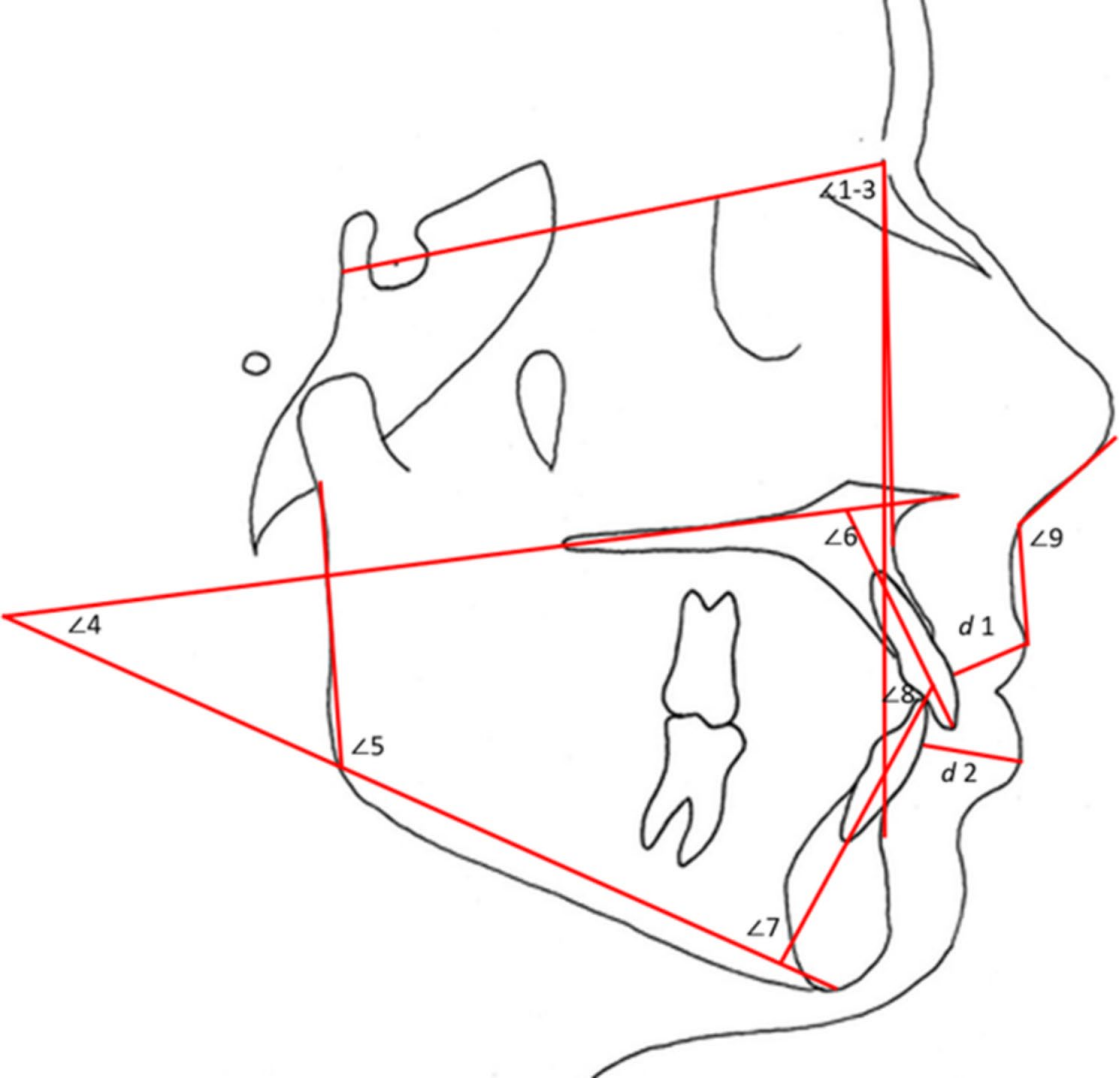
Angular (^o^) and linear (mm) measurements used in the study: SNA (∠1), SNB (∠2), ANB (∠3), Maxillary mandibular plane angle (∠4), Gonion angle (∠5), Upper incisor inclination (∠6), Lower incisor inclination (∠7), Interincisal angle (∠8), Nasolabial angle (∠9), Upper lip thickness (d1), and Lower lip thickness (d2)

Thirty-two 2D landmarks were identified on the LCR. The chosen landmarks are commonly used in the traditional metrical and geometric morphometric systems, are familiar to most orthodontists in lateral cephalogram tracing for malocclusion determination, and show significant biological relevance (Fig. 1; Table 1). These landmarks were used to acquire linear and angular measurements (Fig. 2) and quantitatively assess the hard and soft tissue changes after orthodontic treatment.

Digital copies of LCR in JPG format were converted into TPS files via TPSUtil software (Rohlf, 2015). A dataset containing the landmark coordinates (x, y) of all samples was created using TPSDig2 software. This dataset was then exported into MorphoJ software (version 2.0; Apache licence, Klingenberg Lab) for shape analysis.

**Table 1:** Landmarks used for Geometric Morphometric Analysis

No	Name	Description
1	Ab	Anterior border of the ramus
2	Condylion	The most lateral point on the surface of the condyle of the mandible
3	Articulare	The point of intersection of the external dorsal contour of the mandibular condyle and the temporal bone
4	Pb	Posterior border of the mandible between Ar and Go
5	Gonion	The most posterior and inferior point on the bony chin
6	Mandible Point	The instrumentally determined mid-point of the mandible between gonion and menton
7	Menton	The most inferior point of the mandibular symphysis
8	Pogonoin	The most prominent point of the mandibular symphysis
9	B-Point	Deepest point on the curved profile of the mandible between the chin and alveolar crest
10	Mn Anterior Infradentale	Highest anterior point of the mandibular alveolar bone
11	LiT	Incisor tip of mandibular incisors
12	LiA	Root Apex of mandibular incisors
13	Mn Posterior Infradentale	Highest posterior point of the mandibular alveolar bone
14	Pg’	The most prominent point of posterior symphysis
15	UiT	Incisal edge of upper central incisors
16	UiA	Root Apex of maxillary incisors
17	Prosthion	The point of the upper alveolar process that projects most anteriorly
18	A-Point	The deepest point on the contour of the maxillary alveolar process
19	ANS	Anterior nasal spine
20	PNS	Posterior nasal spine
21	SAHP	The most superior and anterior points of the hard palate
22	Mx Posterior Infradentale	The intersection of the alveolar bone of the maxilla with the palatal surface of the maxillary incisor.
23	Pronasale	The most prominent or anterior point of the nose
24	Subnasale	The junction between the lower border of the nose and the beginning of the upper lip
25	Upper vermillion border	The junction between the oral mucosa and the adjacent facial skin of the upper lip
26	Labrale superius	The most anterior point on the margin of the upper membranous lip
27	Lips Contact Point	Contact or the shortest distance between the upper and lower lips
28	Labrale inferius	The most anterior point on the margin of the lower membranous lip
29	Lower vermillion border	The junction between the lower lip vermillion and the facial skin
30	Sulcus inferius	The point of greatest concavity in the midline of the lower lip between the labrale inferius and the soft tissue pogonion
31	Soft tissue Pogonion	The most prominent point of the soft tissue chin
32	Soft tissue Menton	A point on the soft tissue chin from vertical extension from the menton.

### Shape analysis and statistical analysis

For GMA, all shape and statistical analyses were done using MorphoJ software. The hard and soft tissue shape variations were analysed using Generalised Procrustes Analysis (GPA). LCR were superimposed, translated, scaled, and rotated until the minimal sum of squared distances between corresponding landmarks was detected [22]. This provided the general shape of the hard and soft tissues in Class II division 1 malocclusion samples.

The primary outcome of the study was the hard and soft tissue shape variation (T1), obtained by calculating the most significant variation in the dimension of the hard and soft tissue shapes via Principal Component Analysis (PCA). This technique reduces the shape data dimensionality and identifies variation patterns. The covariance matrix is calculated from the shape data, to compute the eigenvectors of the matrix, also known as Principal Components (PC). Each PC represents the directions of the maximum shape variance in descending order [23].

Another primary outcome compared the shapes and changes before (T1) and after (T2) orthodontic treatment. Discriminant Analysis (DA) was done, where a permutation test (1000 random permutations) was used to assess the significant shape differences between repeated measurements of the hard and soft tissues. This is critical to prevent digitizing errors and subsequently introduce variance in the subsequent analysis. DA was also done separately for the maxilla, mandible, and soft tissue for better visualisation. Procrustes distance was used to evaluate the statistical differences at T2.

In addition to GMA, angular and linear measurements were obtained to confirm the changes in DA as per Fig. 2. Dependent t-tests were performed using Statistical Package for the Social Sciences (version 25; SPSS, Chicago, IL) with a set significance level of *p* < 0.05 and a 95% confidence level to appraise the mean changes before and after orthodontic treatment. In the presence of normally distributed data, descriptive statistics were calculated for each measurement before and after orthodontic treatment while significant differences between them were tested with the dependent t-test at T1 and T2.

### Reliability

Inter-examiner and intra-examiner reliability tests were carried out on 20 randomly selected cases 1 month apart. The tests were performed using the Interclass Correlation Coefficients (ICC) test. The correlation coefficients for intra-examiner reliability and inter-examiner reliability were both above 0.97 and 0.94, respectively, demonstrating excellent agreement between measurements. For GMA, measurement error from landmarking was checked with Procrustes ANOVA using MorphoJ software. The measurements were reliable as the individual ANOVA variation was significantly higher in the shape of the centroid (MS = 0.000095) compared to the digitizing error (MS = 0.000017) (*p* < 0.05). In addition, outlier identification was also done in MorphoJ to ensure the robustness of the samples; no outlier was found.

## Results

**Fig. 3:**
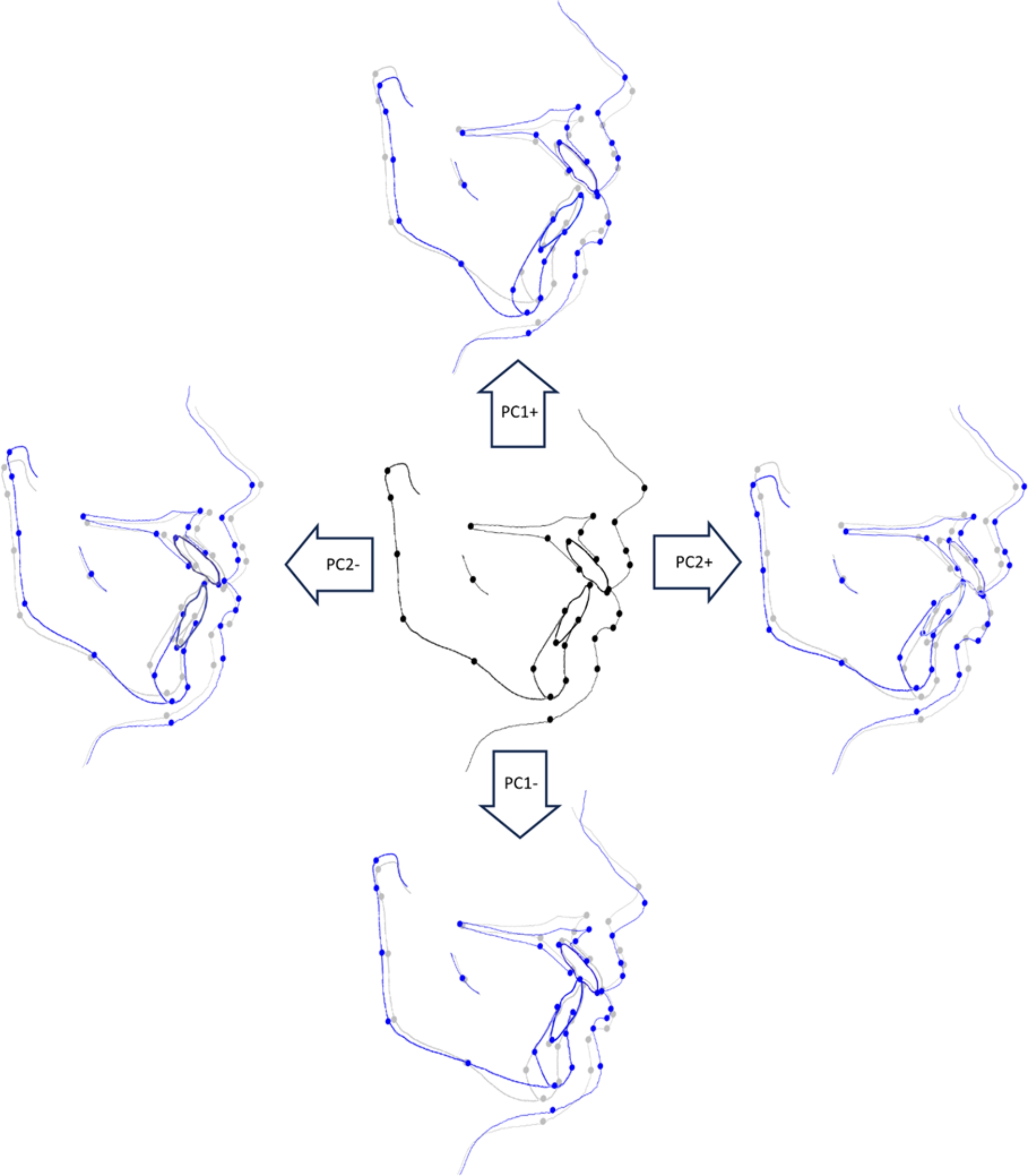
Hard and soft tissue shape variations; black and grey show the mean shape at T1, while blue shows the shape variations associated with PC1 and PC2.

The sample comprised 97 females (69%) and 41 males (31%), with a mean age of 21.88 ± 5.58 years old. The principal component analysis displayed a multivariate analysis and major features in shape variation. In this study, PCA yielded 60 dimensions of hard and soft tissue shape variations, represented by PC1 to PC60. PC1 and PC2 represent the two highest variations within the whole data (Fig. 3).

PC1 (23.7%) shows the highest variations and describes them in the vertical dimension; the facial profile varies from hypodivergent to hyperdivergent. The gonion angle and the maxilla and mandibular plane angles vary from acute to obtuse. The upper lip lengths vary from long to short. PC2 (16.5%), on the other hand, describes variations in the anteroposterior dimension, where the severity of the Class II skeletal pattern varies from mild to severe. The facial profile varies from straight to convex, while lip protuberance varies from everted to inverted (Fig. 3).

**Fig. 4:**
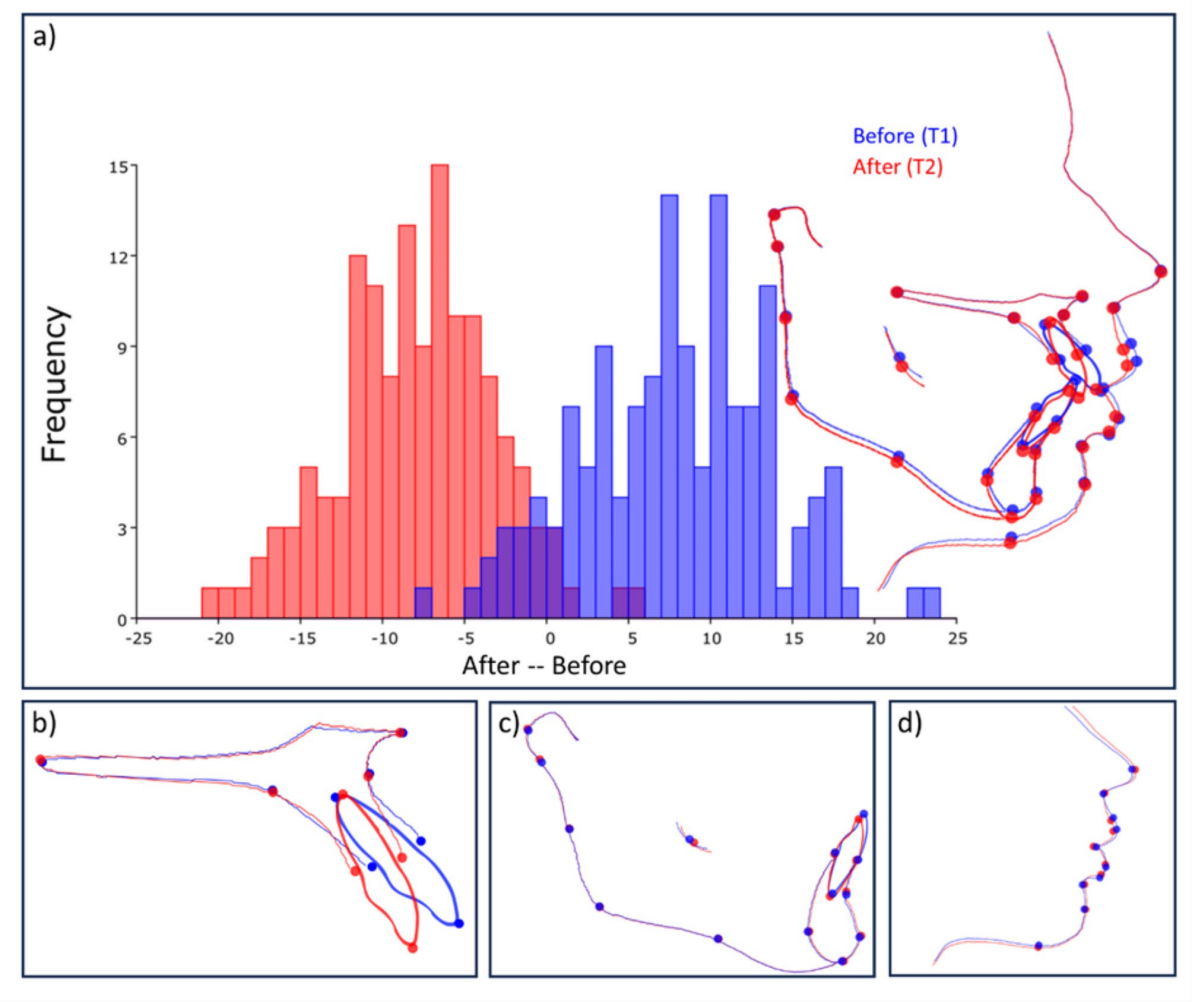
Discriminant analysis (DA) of hard and soft tissue; 4(**a**): Analysis graph and warped outline drawing before (T1) and after orthodontic treatment (T2); 4(**b**): DA of the maxilla; 4(**c**): DA of the mandible; and 4(**d**): DA of the soft tissues

Figure 4 shows the DA of hard and soft tissues before (T1) and after (T2) orthodontic treatment. The Procrustes distance between the mean shapes of T1 and T2 was 0.045. The permutation test showed significant shape variations between T1 and T2 (*p* < 0.0005). After orthodontic treatment, observable significant changes included an increase in the interincisal angle (17.53^o^, *p* < 0.0005). There was also a reduction in the SNA (0.66^o^), SNB (0.03^o^), and ANB (0.64^o^), but an increase in MMPA (0.45^o^). However, these changes were not statistically significant (*p* > 0.05) (Table 2; Fig. 4a). The permutation test showed a significant shape variation between T1 and T2 (*p* < 0.0005) in the maxilla, with a Procrustes distance of 0.105. After treatment, significant retroclination of the upper incisors (17.5^o^, *p* < 0.0005) and remodelling of the alveolar bone following the incisors were observed (Fig. 4b). A significant shape variation was also seen in the mandible (*p* < 0.0005) with a Procrustes distance of 0.021. Lower incisors were uprighted by 3.73^o^ (*p* < 0.0005), and there was intrusion of the lower incisors with downward remodelling of the B point (Fig. 4c). The permutation test showed a significant shape variation (*p* < 0.0005) in the soft tissue, with a Procrustes distance of 0.045. The NLA significantly increased by 8^o^ (*p* < 0.0005), and the upper lip thickness increased by 1.55 mm (*p* < 0.0005). However, the lower lip thickness reduced significantly by 0.77 mm (*p* < 0.0005). A reduction of interlabial distance was also observed (Fig. 4d).

**Table 2:** Linear and angular measurements used in this study

Measurement		Pre-treatment (T1) mean ± SD (^0^)			Near-End- Treatment (T2) mean ± SD (^0^)			Mean differences	*p*-value
Angular Measurement	SNA	82.71	±	3.51	82.04	±	3.63	-0.66	0.221
	SNB	77.24	±	3.47	77.21	±	3.53	-0.03	0.336
	ANB	5.46	±	1.28	4.82	±	1.99	-0.64	0.662
	UiMx	126.82	±	8.73	109.27	±	8.40	-17.53	0.000*
	LiMn	100.18	±	7.76	96.50	±	7.65	-3.73	0.000*
	IIA	105.03	±	9.36	123.86	±	9.19	20.80	0.000*
	GA	125.02	±	6.81	124.99	±	6.77	0.01	0.994
	MMPA	27.97	±	6.17	28.42	±	6.50	0.45	0.548
	NLA	86.70	±	13.83	94.88	±	13.61	8.00	0.000*
Linear Measurement	ULT	11.48	±	1.63	13.05	±	1.97	1.55	0.000*
	LLT	15.15	±	1.90	14.38	±	2.07	-0.77	0.000*

*Significant level of *p* < 0.05 for dependent t-tests

## Discussion

The highest variation (PC1) was observed in the vertical dimension, which showed hypodivergent and hyperdivergent facial profiles. This finding aligns with other studies showing that Class II division 1 maloclusion is associated with a range of vertical skeletal patterns [24]. The second highest variation (PC2) was seen anteroposteriorly, indicating a mild to severe Class II skeletal pattern. This variation may be contributed by prognathic maxilla, retrognathic mandible, or a combination of both [24]. Sassouni [25] stated that two basic types of profile disproportions exist in the Class II skeletal pattern: vertical deviations and anteroposterior deviations. Facial types and deformities may be derived from a combination of vertical and anteroposterior deviations; the identification of these facial types is important for determining a treatment plan since the orthodontic mechanics and considerations used to treat patients are heavily influenced by underlying skeletal discrepancies [26]. This information helps orthodontists understand the shape variations in order to diagnose and formulate an accurate orthodontic treatment plan. For example, the variation in the anteroposterior dimension shows the severity levels of the Class II skeletal pattern (mild to severe), where treatments vary from orthodontic camouflage, growth modification, to orthognathic surgery [27]. The vertical variation also influences the complexity of the treatment plan and mechanics. An orthodontist must treat hyperdivergent cases extra cautiously due to the high anchorage demand in Class II division 1 malocclusion, as anchorage loss may occur more rapidly [5]. Hence, correct extraction patterns and anchorage planning are crucial prior to treatment to ensure successful outcomes. In addition, hyperdivergent cases usually require the intrusion of posterior teeth [28]. Therefore, the orthodontist must master the use of the mini-screw and its mechanics, given its inherent complexity and lack of predictability. Growth modification in a hyperdivergent case is challenging, as it can worsen the overbite even further [29]. Hence, close monitoring and regular follow-ups are essential for treatment. On the other hand, hypodivergent cases usually have an increased overbite, which can also be challenging at times. Flattening of the curve of Spee and extrusion of the premolars or molars can help reduce the overbite. This increases the MMPA and LAFH, ultimately improving the facial profile after orthodontic treatment [30].

While Class II division 1 malocclusion are usually presented with protruded and incompetent lips, the present study indicates that the soft tissue lips can also be everted, long or short. The relationship between the hard and soft tissue profiles varies because some soft tissue structures are closely related to those of hard tissues, while others are influenced by their length, thickness, and function [31].

Morphometric analysis has been shown to be reliable in detecting skeletal, dental, and soft tissue shapes. Skeletally, DA showed a slight reduction of the SNA and SNB, while dentally, there was uprighting of both upper and lower incisors. Proclined incisors, such as those in Class II division 1 malocclusion, are often treated by extraction of premolars and retraction of anterior teeth with anchorage. It is often believed that alveolar bone remodelling occurs spontaneously with orthodontic tooth movements. When the incisors are retracted more posteriorly, the alveolar bone will be retracted as well, causing a reduction in SNA and SNB value [32]. A study by Sharma [33] discovered a retraction of upper and lower incisors, resulting in a reduction of 2.3° and 1.9° for SNA and SNB, respectively. The present study’s linear and angular measurements showed a similar reduction of SNA and SNB by 0.66° and 0.03°, respectively, though the changes were smaller.

The present study observed a significant reduction in the inclination of the upper and lower incisors, together with an increase in the interincisal angle. The upper incisor uprighting in Class II division 1 malocclusion was in line with other studies, where the reduction of the upper incisor inclination varies from 7.9° to 25° [21, 34, 35]. Extractions of the upper first premolars are often performed in this malocclusion [31], as they enable the retraction of the upper incisors, the reduction of the overjet, and the correction of the proclined incisors to a normal inclination [36].

When the lower arch is well aligned with a flat curve of Spee and an increased overjet, extractions may be limited only to the upper premolars. However, when extraction is warranted in crowding cases, second premolars are preferred in the lower arch to preserve the position of the lower incisors, which helps overjet reduction. It is also believed that extractions of the first mandibular premolars produce more incisor retractions, whereas extractions of the second mandibular premolars enable more mesial movement of the first mandibular molars [37]. In this study, the lower incisor inclination decreased by only 3.73°, which was much less compared to that of the upper incisors. These findings are similar to those discovered by Shearn and Woods [38], where the lower incisor inclination was reduced by only 2.9° ± 9.1°. The uprighting of the upper and lower incisors contributed to the significant increase in the interincisal angle by 17.5°, in line with a study that discovered a 12.9° increase in the interincisal angle [39]. To ensure good stability of the overbite after an orthodontic treatment, achieving an occlusion stop of the upper and lower incisors with a good interincisal angle is important. Other studies have suggested that achieving an interincisal angle between 135° and 140° can inhibit incisor overeruption and thus have an impact on the stability of a deep bite [40].

While orthodontic treatment resulted in an increase in the MMPA, the observed effects were not significant. Differential outcomes have resulted from theories regarding the impact of orthodontic treatment on MMPA, or vertical height. This can be explained by the extrusion of the molars or a “wedging effect,” especially when Class II elastics were extensively used [41]. The use of Class II elastics is essential in this malocclusion as it causes retroclination of upper incisors, proclination of lower incisors, retraction of the maxilla, anterior movement of the mandible, and mesialization with extrusion of lower molars in a clockwise rotation of the occlusal plane, which leads to an increase in MMPA and LAFH [42]. Nevertheless, the use of Class II elastics in hyperdivergent cases must be well monitored to prevent worsening of malocclusion. The mechanics of levelling the curve of Spee will mainly cause extrusion of lower premolars, proclination of the lower incisors, extrusion of molars, and a clockwise rotation of the occlusal plane, thus causing an increase in LAFH [43]. This effect could be significant, as Class II division 1 malocclusion showed the deepest curve of Spee compared to other malocclusions [44].

Many studies have also attempted to quantify the effect of upper incisor retraction on the lips. In the present study, a flattening of the lips at T2 was observed with the retraction of the upper incisors, accompanied by the upper lip retraction at a mean ratio of 2:1. Kasai [31] stated that every 4.3 mm on the upper incisor retraction would result in a 1.9 mm upper lip retraction, whereas Talass et al. [45] found that the ratio of the upper incisor retraction to the upper lip retraction is 4 to 3. For lip thickness, the predictive accuracy of post-orthodontic treatment using lateral cephalometric radiographs is still poor. As a general rule, retraction of the upper incisors will eliminate lip strains and cause an increase in lip thickness; if the retraction of incisors continues, there will be a retraction of the upper lip as well [46]. This is because soft tissue changes are easily affected by incisor movements, soft tissue thickness, lip strains, and the presence of a lip trap [47]. This explains the increase in upper lip thickness in the present study, which is similar to the findings by Lai, Ghosh, and Nanda [48], where the thickness of the upper lip increased and the thickness of the lower lip decreased after incisor retraction. In addition, there was a significant reduction in the interlabial distance and an increase in NLA. This aligns with findings by Jacob and Buschang [49], where for every 1 mm of maxillary incisor retraction, there was a 0.5 mm reduction in the interlabial gap, though they were uncertain of the mechanism by which this occurred. Some studies conclude that the change is primarily due to the inferior movement of the upper lip, while others state that interlabial gap reduction is due to a lengthening of the lower lip [31, 45]. Regardless, there is an agreement that if the incisors are retracted, there is lengthening of one or both lips. Concurrently, the inferior and retraction movements of the upper lip adapting to the new inclination or upright incisors, increases the NLA after orthodontic treatment. Talass et al. [45] stated that a greater increase in NLA occurs with the presence of thicker soft tissues subnasale before treatment, a greater amount of upper incisor edge retractions, a thinner upper lip, a smaller overjet before treatment, and an increase in the lower facial height following orthodontic treatment.

In orthodontics, GMA has been found to be more beneficial for investigating and describing complex hard and soft tissue shapes compared to traditional analysis [50]. Recently, it has been used effectively to assess the shape differences in various growth patterns [11], age differences [51], genders [52], soft tissues [16], and shapes of mandibular symphysis in different malocclusions [17, 50] without additional radiation exposure. The present study offers insights into potential future research avenues, including 3D analysis, different malocclusions, and the comparison of age and ethnicity. However, it should be noted that these were the limitations of this study. The use of GMA in this study also provides much more soft tissue information by fully utilising the lateral cephalometric radiographs that were already in use for routine orthodontic treatment. It has been recognised that as some areas of soft tissue follow skeletal change, not all parts of the soft tissue directly follow the underlying skeletal profile [53]. Currently, the paradigm shift has placed priority on soft tissue treatment over hard tissue results [54], and orthodontic treatment requirements are primarily aesthetic requests [54]. Therefore, recognising the effect of orthodontic treatment on the soft tissue is equally, if not more, important during diagnosis to evaluate the patient’s perceived aesthetics. This shows that analysis that incorporates both hard and soft tissue analysis such as GMA will enable orthodontists to fully understand the effect of orthodontic treatment, diagnose appropriately, and formulate a customised treatment plan. In addition, doctor-patient communication can be enhanced, thus providing holistic management without overestimating treatment outcomes or underestimating the complexity of procedures.

## Conclusion

1. Skeletally, the facial profile varies from hypodivergent to hyperdivergent, as well as mild to severe skeletal II.
2. The incisor inclination varies from upright to procline, and the alveolar bone height ranges from short to long.
3. The nasolabial angle varies from acute to obtuse. The upper lip length varies from long to short, while the thickness of both upper and lower lips varies from thick to thin, as well as everted or inverted.
4. After orthodontic treatment, the upper and lower incisors become significantly upright. The interincisal angle, NLA, and the upper lip thickness increase, whereas the lower lip thickness reduces significantly.
5. GMA improves the diagnostic accuracy of lateral cephalometric radiographs in orthodontic treatment by enabling the identification and description of complex shapes and changes.

### Electronic supplementary material

Below is the link to the electronic supplementary material.

Supplementary Material 1

Supplementary Material 2

## Data Availability

The datasets used and/or analysed during the current study are available from the corresponding author on reasonable request.
